# A Comparative Analysis of Commonly Used Surgical Approaches for Anterior Acetabular Fractures

**DOI:** 10.7759/cureus.38979

**Published:** 2023-05-13

**Authors:** Ajay Sharma, Surabhi Das, Raghavendra Kaganur, Nirvin Paul, J Pragadeeshwaran, Chandra K khande, Bom Bahadur Kunwar

**Affiliations:** 1 Trauma Surgery and Critical Care, All India Institute of Medical Sciences, Rishikesh, Rishikesh, IND; 2 Orthopaedics, All India Institute of Medical Sciences, Rishikesh, Rishikesh, IND; 3 Trauma Surgery, All India Institute of Medical Sciences, Rishikesh, Rishikesh, IND

**Keywords:** ilioinguinal approach, stoppa approach, anterior wall, acetabulum, anterior column

## Abstract

Introduction

Fractures of the acetabulum are inherently complex due to the anatomy of the innominate bones and also the presence of several vital neurovascular structures in the vicinity. Thus, the treatment of pelvic ring and acetabulum fractures is riddled with complexities and is considered among the most challenging surgeries for an orthopedic surgeon.

When anterior access is necessary, such as in the anterior column, both columns, anterior column posterior hemitransverse, transverse, and T-type fractures, both the ilioinguinal and the anterior intrapelvic (AIP) or modified Rives-Stoppa methods are employed. The aim of this study is to compare the results from acetabular fractures treated with a modified Stoppa and ilioinguinal technique.

Materials and methods

We conducted a prospective cohort study to compare the outcomes of anterior acetabular fracture fixation using the modified Stoppa approach and the ilioinguinal approach. The outcomes measured were the amount of intraoperative bleeding, surgery duration, postoperative quality of fracture reduction, postoperative drain collection, and postoperative neurovascular status.

The functional outcome was measured at three, six, and 12 months using the Merle d'Aubigné score. The radiological outcome was measured using the Matta scoring system.

Results

A significant difference was noticed in the two groups in the average blood loss and surgical duration, where the mean blood loss was 911.67 ± 143.05 ml in the ilioinguinal approach and 748.33 ± 165.30 ml in the modified Stoppa approach. While the ilioinguinal approach had a mean surgical duration of 190.33 ± 29.42 minutes, the modified Stoppa approach had 151.33 ± 23 minutes. The difference in postoperative fracture reduction in both groups was insignificant. The lateral femoral cutaneous nerve was compromised in 8.33% of cases in group A. The obturator nerve was compromised in 6.67% of cases in group B. The postoperative functional outcome was assessed by the modified Merle d’Aubigné score, and the radiological outcome was evaluated by the Matta score. The results obtained in both our study arms were comparable.

Conclusion

Based on our results, we can safely advocate the superiority of the Stoppa approach over a more extensive ilioinguinal approach. By virtue of being shorter in surgical duration and causing lesser blood loss, the Stoppa approach seems to be a better alternative, especially in elderly or polytrauma patients.

As no difference was noted in the postoperative outcomes both clinically and radiologically, no approach showed superiority over the other in terms of patients’ eventual functional outcomes.

## Introduction

Fractures of the acetabulum are inherently complex due to the anatomy of the innominate bones and the presence of several vital neurovascular structures in the vicinity. Thus, the treatment of fractures of the pelvic ring and acetabulum is riddled with complexities and is considered one of the most challenging surgeries for an orthopedic surgeon. Selection of the appropriate approach is of paramount importance to achieve acceptable anatomical reduction and avoid complications, some of which could be fatal.

There are multiple surgical approaches for the surgical fixation of acetabular fractures. Choosing an approach requires a thorough understanding of the fracture pattern as no single approach allows access to the entire acetabulum. For the internal repair of fractures of the acetabulum and pelvic ring, Judet and Letournel devised the ilioinguinal technique [1-3]. The most frequent reasons for its usage are the management of anterior column and wall fractures, related anterior column and posterior hemitransverse fractures, specific both-column fractures, and rarely T-shaped and transverse fracture patterns. The anterior portion of the sacroiliac joint to the pubic symphysis, as well as the quadrilateral surface, are almost completely exposed, giving access to the inner aspect of the innominate bone [3-6]. The possibility of injury to the external iliac vessels and femoral nerve poses a difficulty since they are encountered in the surgical plane during the approach, making it a laborious exposure [3].

Stoppa et al. [7] described the Stoppa approach as a subperitoneal median technique for the treatment of groin hernias first in 1973. In the early 1990s, the approach was introduced as the method for approaching the pelvic bone and anterior acetabulum by Hirvensalo et al. [8] and Cole and Bolhofner [9]. With the extra danger of vascular bleeding from the corona mortis or the obturator veins, this approach uses a single window to obtain an intrapelvic overview of the surgical field while keeping the entire peritoneal sac and its contents away from the fracture site. However, this method does not allow for the fractures to be moved in order to reduce the iliac wing anatomically, which is a critical step in reducing the anterior column anatomically.

Consequently, a second lateral iliac wing window is required in addition to the Stoppa method. Jakob et al. [10] and Andersen et al. [11] described a modified approach using the Stoppa and iliac surgical window for managing acetabular fractures.

When anterior access is necessary, such as in the anterior column, both columns, anterior column posterior hemitransverse, transverse, and T-type fractures, both the ilioinguinal and the anterior intrapelvic (AIP) or modified Rives-Stoppa methods are employed [3,11,12]. The aim of this study is to compare the results from acetabular fractures treated with a modified Stoppa and ilioinguinal technique.

## Materials and methods

We devised a prospective cohort study to compare the outcomes of anterior acetabular fracture fixation using the modified Stoppa approach and the ilioinguinal approach. The outcomes measured were the amount of intraoperative bleeding, surgery duration, postoperative quality of fracture reduction, postoperative drain collection, and postoperative neurovascular status. The sample size was calculated at a 95% confidence interval and an alpha error of 0.05 assuming a standard deviation of 48.79 minutes in the operative time between two procedures [13]. To detect a minimum difference of 35 minutes in the operative time between the two approaches at a study power of 80%, the required sample size was 60. This study was approved by the institutional review board.

Patients with acetabular fractures that were considered to be fully treated preoperatively through an anterior intrapelvic approach, such as anterior column fractures, anterior wall fractures, the anterior column with posterior hemitransverse fractures, some fractures associated with columns, partial transverse fractures, and partial T-type fractures, were included, provided they gave written informed consent for the procedure. Exclusion criteria included patients with a pre-existing hip disease on the same side, which could create bias in the clinical-radiological evaluation of the results. Additionally, patients who solely required the Kocher-Langenbeck technique for fixation because of a displaced posterior column or posterior wall fracture were not included in the study. Sixty patients conforming to the inclusion and exclusion criteria, who presented to the emergency bay of our tertiary care hospital between April 2020 and June 2021, were recruited. They were treated by either the modified Stoppa approach or the ilioinguinal approach based on the involvement of the quadrilateral plate, where the Stoppa approach was preferred. Twenty-eight patients were treated by the modified Stoppa approach and 32 patients by the ilioinguinal approach.

Preoperative planning

Adequate radiographs of the pelvis were taken, with bilateral hips anteroposterior (AP) view along with iliac oblique and obturator oblique views of the affected side. Computed tomography (CT) scan with three-dimensional (3-D) reconstruction was obtained for preoperative planning in addition to the roentgenogram.

Surgical approaches

Group 1 (Ilioinguinal Approach)

**Figure 1:**
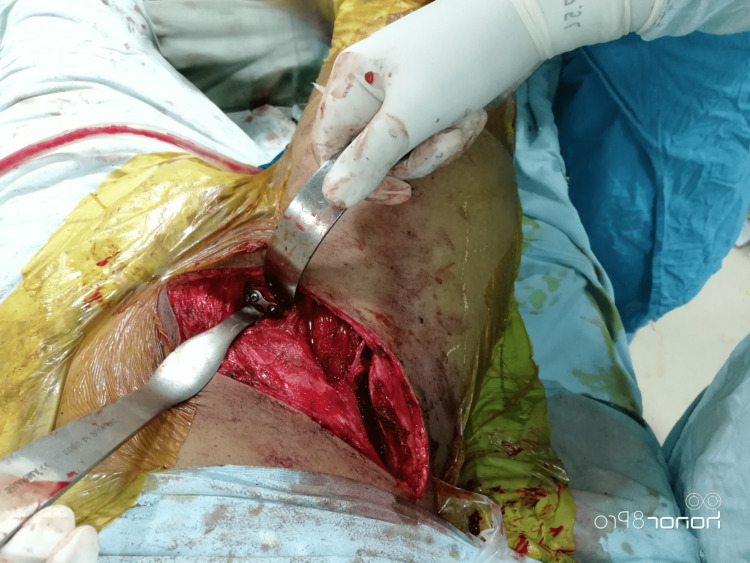
Ilioinguinal approach

The patient was placed in the supine position. An incision was made from two fingerbreadths above the symphysis pubis up to the anterior superior iliac spine (ASIS) and curved along the iliac crest. Abdominal muscles were released from the iliac crest, and the inner table was exposed by blunt dissection. External oblique aponeurosis was incised in line with the skin incision. The spermatic cord or round ligament was identified and protected. The inguinal ligament was split away from the main portion of the ligament taking care not to injure the external iliac vessels that lie under the medial portion of the ligament and the lateral femoral cutaneous nerve that lies under the lateral portion of the inguinal ligament. The femoral neurovascular structures were exposed in the middle of the surgical wound. The iliopectineal fascia lying between the iliopsoas muscle and the vessels was divided down to the pelvic brim and then cut proximally along the brim. Three windows were formed: a lateral window lying lateral to the iliopsoas and exposing the iliac fossa and the pelvic brim, a middle window in between the iliopsoas muscle and the external iliac vessels providing access to the pelvic brim and the quadrilateral surface, and a medial window medial to the vessels and exposing the pubis and the superior pubic ramus (Figure 1).

Group 2 (Modified Stoppa Approach)

**Figure 2:**
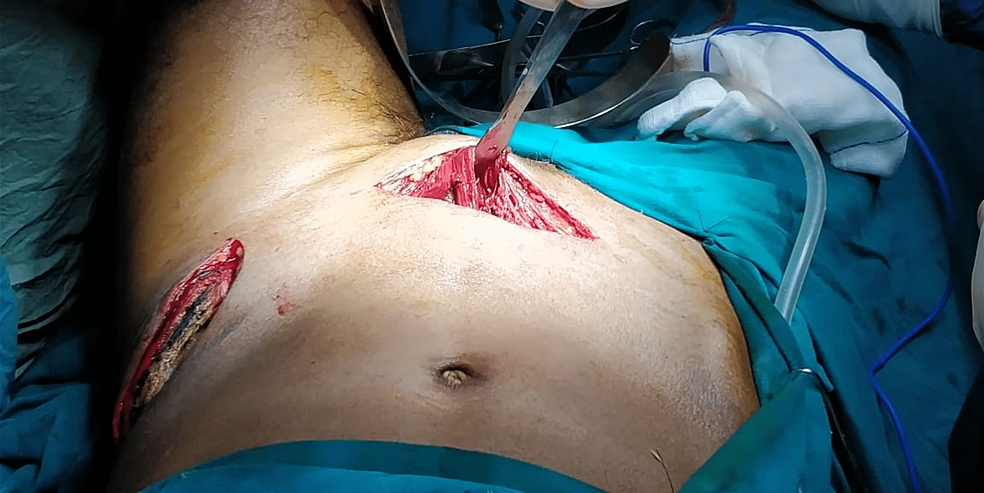
Modified Stoppa approach

The patient was placed in the supine position with a bump under the knee to relax the iliopsoas. A transverse incision was made above the symphysis pubis, then the linea alba was incised, and the ipsilateral rectus abdominis was divided from the superior ramus. As the dissection was extended toward the acetabulum, the corona mortis encountered was ligated, and the dissection was further advanced along the pelvic brim. The iliopectineal fascia was detached, and retractors were placed under the iliopsoas. The quadrilateral surface was exposed, and a blunt Hohmann retractor was placed between the quadrilateral surface and the internal obturator muscle into the sciatic notch. The lateral window was made to aid the reduction and fixation of fractures exiting through the iliac crest (Figure 2).

Perioperative care

All surgeries were performed by two senior surgeons who had pelviacetabular trauma training. All 60 patients received the same perioperative care. They were administered third-generation cephalosporin preoperatively 12 hours before surgery, which was continued for 48 hours in the postoperative period and was supplemented by gram-negative coverage. All patients were prescribed oral antibiotics for another five days after discharge. Drain removal was done on the second postoperative day. The patient was discharged on postoperative day 3 if the immediate postoperative period was unremarkable. Indomethacin 25 mg thrice daily was prescribed orally for six weeks from the day after surgery. Sitting was allowed along with calf muscle stretching and contraction, knee bending, and quadriceps exercise from the second day. Suture removal was done after three weeks, and a passive range of motion was started. Toe touch weight bearing was allowed at eight weeks postop along with active abduction, adduction, and flexion strengthening exercises.

Follow-up

All the patients were followed up for a minimum of one year, at three monthly intervals.

Outcome measures

Patients were evaluated using functional, radiological, and general outcome measures. The functional outcome was measured at three, six, and 12 months using the Merle d'Aubigné score, wherein the outcome was considered excellent with 18 points, good with 15-17 points, fair with 12-14 points, and poor with 3-11 points. The Matta criteria for fracture reduction was used to assess the quality of reduction: anatomic (0-2 mm), imperfect (2-3 mm), and poor (>3 mm). The radiological outcome was measured using the Matta scoring system in which the results were graded as follows [3]: excellent - anatomical or near normal appearance of the hip joint; good - mild changes, small osteophytes, moderate (1 mm) narrowing of the joint, and minimum sclerosis; fair - moderate osteophytes, moderate (<50%) narrowing of the joint, and moderate sclerosis; and poor - advanced changes, large osteophytes, severe (>50%) narrowing of the joint, and collapse or wear of the femoral head and acetabulum.

Statistical analysis

Data were collected by two independent observers, and analysis was done by a third blinded observer. Data were entered in an MS Excel sheet (Microsoft Corporation, Redmond, Washington). Categorical data were compared using the Chi-square test, while parametric data were compared using the unpaired student t-test, and a p-value of <0.05 was considered significant. Statistical analysis was done using Statistical Package for the Social Sciences (SPSS) software, version 26 (IBM Corp., Armonk, NY).

Ethical concerns

Written informed consent was taken from all the patients in their vernacular language before the operation for inclusion in the study. Institutional Review Board (IRB) approval was obtained on December 10, 2019, with IRB number - 785/MC/EC/2019 from the office of the ethics committee of Sawai Man Singh Medical College and attached hospitals, Jaipur, Rajasthan, India.

## Results

A total of 60 patients with acetabulum fractures who were considered to be amenable to treatment with an anterior approach (ilioinguinal or modified Stoppa) were selected for the study. Thirty-two patients were treated via the ilioinguinal approach and were denoted as group A, and 28 patients were treated via the modified Stoppa approach (denoted as group B). The majority of the study population belonged to the age group of 20-40 years (55%), followed by 40-60 years (25%). In our study, the mean age of the study population was 33.43 ± 8.32 years (Table 1).

**Table 1:** General demographic features RTA: Road traffic accident. p-value < 0.05 is considered significant.

	Ilioinguinal Approach (n = 32)		Modified Stoppa Approach (n = 28)		p-value
	n	%	n	%	
Age (years)					0.369
<20	2	6.25	2	7.14	
20-40	17	53.12	16	57.14	
40-60	8	25	7	25	
>60	5	15.622	3	10.72	
Sex					0.041
Male	25	78.12	21	75	
Female	7	21.88	7	25	
Mode of injury					1.000
RTA	26	81.25	24	85.72	
Fall from height	6	18.75	4	14.28	
Side of injury					1.000
Right	17	53.12	15	53.57	
Left	15	46.88	13	46.43	
Fracture pattern					0.675
Anterior column	12	37.50	10	37.71	
Anterior column + anterior wall	10	31.25	7	25.00	
Anterior column + posterior hemitransverse	2	6.25	3	10.72	
Anterior wall	5	15.62	6	21.43	
Transverse	3	9.38	2	7.14	
Associated injuries					0.743
Head injury	2	6.25	1	3.13	
Upper limb injury	1	3.13	0	0	
Lower limb injury	2	6.25	2	6.25	
Abdominal/pelvic injury	2	6.25	2	6.25	

In our study 76.67% of the population were males. Road traffic accidents (RTAs) accounted for 83.33%, whereas 16.67% were due to falls from height. There were no associated injuries in 80% of the study group. About 6.7% had associated lower limb injuries, 8.4% had associated abdominal/pelvic injuries, and 4.9% had associated head injuries. The preoperative neurovascular status was intact in all patients of the study population.

We classified acetabular fractures according to the Letournel classification based on X-ray/CT findings. About 36.67% of the population had anterior column fracture (20% in group A and 16.67% in group B), 28.33% had an anterior column with anterior wall fracture (16.67% in group A and 11.66% in group B), 8.33% had anterior column + posterior hemitransverse (3.33% in group A and 5% in group B), 8.33% had transverse T-type fractures (5% in group A and 3.33% in group B), and 18.33% had anterior wall fracture (8.33% in group A and 10% in group B).

A significant difference was noticed in the average blood loss and surgical duration between the two approaches, where the mean blood loss was 911.67 ± 143.05 ml in the ilioinguinal approach and 748.33 ± 165.30 ml in the modified Stoppa approach. While the ilioinguinal approach had a mean surgical duration of 190.33 ± 29.42 minutes, the modified Stoppa approach had 151.33 ± 23 minutes (Table 2). The difference in postoperative fracture reduction in both groups was insignificant (Table 3).

**Table 2:** Perioperative outcomes p-value < 0.05 is considered significant.

Parameters	Ilioinguinal Approach (n = 32)		Modified Stoppa Approach (n = 28)		p-value
Type of Anesthesia	n	%	n	%	
General	5	15.62	4	14.28	1.000
Spinal	27	84.38	24	85.72	
Mean blood loss (ml)	911.67 ± 143.05		748.33 ± 165.30		<0.001
Mean surgical duration	190.33 ± 29.42		151.33 ± 23.00		<0.001
Wound drainage (ml/day)	141.17 ± 27.09		123.33 ± 45.93		0.105

**Table 3:** Quality of reduction p-value < 0.05 is considered significant.

Quality of Reduction	Ilioinguinal Approach (n = 32)	Modified Stoppa Approach (n = 28)	Total	p-value
Anatomic	11	10	21	0.653
Imperfect	15	13	28	
Poor	6	5	11	

The lateral femoral cutaneous nerve was compromised in 8.33% of cases in group A. The obturator nerve was compromised in 6.67% of cases in group B. Five patients with the ilioinguinal approach and three with the modified Stoppa approach developed a superficial infection, which was managed with antibiotics. No inguinal hernias were seen in our study (Table 4).

**Table 4:** Postoperative complications p-value < 0.05 is considered significant.

	Ilioinguinal Approach (n = 32)	Modified Stoppa Approach (n = 28)	p-value
Neurovascular injury	5	4	0.831
Obturator nerve	0	4	
Lateral femoral cutaneous nerve	5	0	
Wound infection	5	3	0.704

Postoperative functional outcome was assessed by modified Merle d'Aubigné score (Table 5), and radiological outcome was evaluated by Matta score (Table 6). The results obtained in both our study arms were comparable.

**Table 5:** Postoperative follow-up Merle d’Aubigné score at six months NA: Not applicable. p-value < 0.05 is considered significant.

	Merle d'Aubigné Score								
	At one month			At 3 months			At 6 months		
	Excellent	Good	Fair	Excellent	Good	Fair	Excellent	Good	Fair
Modified Stoppa approach	0	0	28	0	10	18	8	14	6
Ilioinguinal approach	0	0	32	0	14	18	9	17	6
P-value	NA			0.279			0.830		

**Table 6:** Matta radiological outcome score p-value < 0.05 is considered significant.

	Excellent	Good	Fair
Modified Stoppa approach	13	15	4
Ilioinguinal approach	11	4	3
P-value	0.564		

## Discussion

Since the groundbreaking work of Letournel and Judet, acetabular surgery has advanced significantly as have all orthopedic treatments. With the advent of good outcomes with the modified Stoppa approach, there is an ongoing debate regarding the optimal approach for managing anterior acetabular fractures [8].

Acetabular fractures are most commonly seen in young males due to their propensity for being victims of high-velocity RTAs [14]. However, over the past few years, the incidence of acetabular fractures in the geriatric population has risen steeply [15]. In this study, we found the incidence to be higher in young males, with few patients from the geriatric population. The most common mode of injury seen in our study was RTA, which correlated with other studies by Cole et al. [9] and Adawy et al. [16]. The majority of the patients were male, which was in concordance with the available literature [11,16-19].

The ilioinguinal approach has been a standard approach for the treatment of acetabular fractures over the last few decades, with 21.9% of acetabular fractures being treated by this approach historically [20]. This approach is time-consuming, has a long learning curve, has high blood loss, and so on. Additionally, it is inherently dangerous for femoral nerves and external iliac arteries. The combined view through the three windows (medial, middle, and lateral) in this approach permits a limited view and evaluation of the fracture; hence, indirect reduction maneuvers are frequently used. Moreover, the ilioinguinal approach being extensive is associated with a score of complications, i.e., lymphoedema, hematoma, hernias, thrombosis, lesions of the femoral vessels, and wound healing problems [21]. The aforementioned complications are less likely to occur in the hands of experienced surgeons, but there is still an inherent danger of injuries to the neurovascular injuries [22].

The modified Stoppa approach, an anterior intrapelvic approach to the anterior acetabulum, has also gained popularity over the last few years. It is a modification of the Rives and Stoppa approach for the repair of inguinal hernias using a Dacron graft [7], with continued dissection laterally and posteriorly along the pelvic brim toward the internal iliac fossa, to reach the acetabulum. It was potentially less invasive and also allowed better visualization of the quadrilateral plate, the entire anterior pelvic ring, and the posterior column [12]. Major complications include obturator nerve injury, superior gluteal artery injury, rectus atrophy, inguinal hernias, and wound complications [12]. It is said to be less time-consuming than the ilioinguinal approach.

We found that the operative duration and the blood loss during surgery of the Stoppa approach were significantly shorter than the ilioinguinal approach. The rate of complications, anatomic reduction, and hip function recovery by the two approaches had no significant statistical difference. In comparison with the ilioinguinal approach, the modified Stoppa approach had significantly lesser intraoperative blood loss and eventually needed less blood transfusion. Time is an important factor in surgery even though it may not be the most important one. Increased tissue stress and wound exposure from longer operations are associated with more consequences, including greater rates of infection, more blood loss, and a higher frequency of both local and systemic issues. Particularly in aged, polytraumatized, or fatigued patients, a longer procedure with more blood loss makes the surgeon's "second hit" stronger, increasing the likelihood of unfavorable results [23].

## Conclusions

Based on our results, we can safely advocate the superiority of the Stoppa approach over a more extensive ilioinguinal approach. By virtue of being shorter in surgical duration and causing lesser blood loss, the Stoppa approach seems to be a better alternative, especially in elderly or polytrauma patients. As no difference was noted in the postoperative outcomes, both clinically and radiologically, no approach showed superiority over the other in terms of patients’ eventual functional outcomes. The limitation of our study is shorter follow-up and small sample size. Hence, large multicentric randomized trials with larger sample sizes are deemed necessary to substantiate our results.
